# Abstracts

**Published:** 1910-04

**Authors:** 


### ABSTRACTS

### CULTIVATE THE READING HABIT.

The demands of an active professional career are so pressing that, unless one is careful to guard against it, he may find himself reading less and less of the current medical literature as his practice is increasing. Yet it is of the utmost importance that this faulty habit be not acquired. One cannot keep abreast of the times in medicine or in other subjects without constant study; and this constant study means a perusal in abstract if not in the original, of the newer ideas that are being constantly advanced. As Edward Jackson, in a recent paper, has so tersely expressed it, "The man who attempts to practise medicine without drawing on the experience of the profession in the past and without contact with the professional thought of his contemporaries remains ignorant and degenerates into the lower type of charlatan." And William Osler has said, "Books are tools, doctors are craftsmen, and so truly as one can measure the development of any particular handicraft by the variety and complexity of its tools, so we have no better means of judging the intelligence of a profession than by its general collection of books. A physician who does not use books and journals, who does not need a library, who does not read one or two of the best weeklies and monthlies, soon sinks to the level of the cross-roads prescriber. The true worker does not want text-books; he looks to journal literature and monographs."

No physician can, with any regard for his profession and his own standing in it, attempt to treat any given class of cases as it was done a decade ago unless he has personal knowledge that no improved methods of treatment have been adopted during that decade; and it is not possible for him to know of such improvements unless he has gained that knowledge from postgraduate work, intimate association with those who have done careful postgraduate work or by careful perusal of the literature during that period. Fortunately for us, there are men who devote a great deal of time and labor to preparing abstracts of the literature along any of these lines. In practically every branch of medicine there are excellent journals that devote a fair proporiton of their pages to such abstract work. By reading one or two general weeklies or monthlies, in order to keep up with what is being done in general medicine, one can devote a considerable portion of his reading time to the perusal of his special journals and the various published abstracts. If, in these abstracts, something is found that is worthy of more than passing notice, something that particularly strikes one's attention, the original can be readily secured and studied at one's leisure. There is no excuse then for not knowing what is going on in the medical world. If one is unable to visit the great medical centers every year or two and learn by direct contact the new methods that are being tried out, he can certainly know of those methods and of the results secured by systematic perusal of the current medical literature. Let us, therefore, cultivate the habit of reading.—*C. A. V., Editorial Northwest Med.*

